# Gentiopicroside inhibits the progression of gastric cancer through modulating EGFR/PI3K/AKT signaling pathway

**DOI:** 10.1186/s40001-024-01637-6

**Published:** 2024-01-11

**Authors:** Qishuai Chen, Tongtong Zhang, Bingjun Li, Zhenguo Zhu, Xiaomin Ma, Yun Zhang, Linchuan Li, Jiankang Zhu, Guangyong Zhang

**Affiliations:** 1 Department of General Surgery, The First Affiliated Hospital of Shandong First Medical University, No. 16766 Jingshi Road, Jinan, 250014 Shandong Province People’s Republic of China; 2 Department of Laboratory Medical, Zibo Central Hospital, Zibo, 255000 Shandong Province People’s Republic of China

**Keywords:** Gentiopicroside, PI3K/AKT signaling pathway, EGFR, Gastric cancer, Apoptosis

## Abstract

**Background:**

This study was designed to clarify the function and potential mechanism of gentiopicroside (GPS) in regulating the malignant progression of gastric cancer (GC) through in vitro cellular experiments and in vivo animal models.

**Methods:**

AGS and HGC27 cells were divided into control group and GPS treatment groups (50 µM and 100 µM). Then, the cellular proliferation, colony formation, migration, invasion, and apoptosis were detected, respectively. Transmission electron microscope (TEM) was used to observe the mitochondrial changes, and the mitochondrial membrane potential (MMP) was determined using the JC-1 commercial kit. Network pharmacology analysis was utilized to screen the potential molecule that may be related to the GPS activity on GC cells, followed by validation tests using Western blot in the presence of specific activator. In addition, xenografted tumor model was established using BALB/c nude mice via subcutaneous injection of HGC27 cells, along with pulmonary metastasis model. Then, the potential effects of GPS on the tumor growth and metastasis were detected by immunohistochemistry (IHC) and HE staining.

**Results:**

GPS inhibited the proliferation, invasion and migration of GC cell lines in a dose-dependent manner. Besides, it could induce mitochondrial apoptosis. Epidermal growth factor receptor (EGFR) may be a potential target for GPS action in GC by network pharmacological analysis. GPS inhibits activation of the EGFR/PI3K/AKT axis by reducing EGFR expression. In vivo experiments indicated that GPS induced significant decrease in tumor volume, and it also inhibited the pulmonary metastasis. For the safety concerns, GPS caused no obvious toxicities to the heart, liver, spleen, lung and kidney tissues. IHC staining confirmed GPS downregulated the activity of EGFR/PI3K/AKT.

**Conclusions:**

Our investigation demonstrated for the first time that GPS could inhibit GC malignant progression by targeting the EGFR/PI3K/AKT signaling pathway. This study indicated that GPS may be serve as a safe anti-tumor drug for further treatment of GC.

## Introduction

Gastric cancer (GC) is still one of the major challenges in the public health [1], newly diagnosed GC each year occur largely in Asian and South American countries [2]. To date, its treatment is highly relied on surgery, chemotherapy, radiotherapy, immunotherapy, as well as targeted therapy, but the GC-related mortality remains high due to frequent metastasis and recurrence [3].

Great attention has been paid to the anti-GC studies based on traditional Chinese medicine (TCM) that are mainly derived from natural products [4]. These drugs have been reported to show anti-cancer effects through inducing apoptosis and inhibiting the proliferation and metastasis of cancer cells [5]. One of an important reason for selecting these TCM agents is their abilities to modulate the cancer-related signaling pathways and molecular targets, without causing adverse events [6, 7]. Recently, GPS, isolated from *Gentianella acuta* with a variety of pharmacological effects, has been utilized for the treatment of cancers, such as ovarian cancer [8], cervical cancer [9] and retinoblastoma [10] as well as GC [11]. However, our understanding on how GPS modulated the GC-related signaling pathways is still limited. In a previous study, Huang et al. reported that GPS regulated the PI3K/AKT signaling pathway based on network pharmacology [11], but their investigations on the molecular mechanisms are not deep.

Epidermal growth factor receptor (EGFR) is abnormally amplified in tumour cases and can be used as one of the targets for cancer therapy [12]. The activation of EGFR triggers a variety of downstream signaling pathways, for such classic pathways including PI3K/AKT/mTOR, ERK/MAPK, which are involved in cell proliferation, cell cycle progression, primary tumourigenesis and metastasis [13]. On this basis, this study was designed to investigate the potential anti-cancer effects of GPS, in which we utilized GC cell lines (i.e., AGS and HGC27) and animal models to analyze the effects of GPS on the cellular proliferation, migration, invasion, apoptosis as well as tumor growth. In addition, we also analysed whether GPS could regulate the PI3K/AKT signaling pathway by inhibiting the expression of EGFR.

## Materials and methods

### Network pharmacology analysis

#### Potential target acquisition

GPS was predicted using SwissTargetPrediction (http://swisstargetprediction.ch) to obtain the target of action of the compounds, and the target genes with probability > 0 were selected, and a total of 136 drug targets were screened.

#### Disease potential target acquisition

Using OMIM (https://www.omim.org/), Genecards (https://www.genecards.org) databases were searched with the keywords (‘gastric cancer’) OR (‘stomach cancer’) OR (‘gastric carcinoma’), respectively. Finally, 5543 disease targets were obtained.

#### Prediction of potential drug-disease targets

Using python for 136 drug targets, 5543 disease targets, drawing the Wayne diagram, the two take the intersection of the common drug-disease targets obtained 64.

#### Protein interaction (PPI) network construction

The drug–disease common targets were uploaded to the database (https://cn.string-db.org/) to obtain the PPI network, and the PPI network TSV file was imported into Cytoscape 3.9.1 software for visualisation. Node size and colour were corresponded to the Degree value of that node (from blue to red with node size from small to large corresponding to Degree value from small to large).

#### KEGG enrichment analysis

The enriched KEGG (http://www.genome.jp/kegg/) pathway data were screened with *P* value < 0.05, and the results of the enrichment analysis were arranged in ascending order of *P* value, and the related pathways ranked in KEGG were selected for visual display.

#### Molecular docking

The PDB files of the 3D structures of the key target proteins were downloaded from the (https://www.rcsb.org/) database and were dehydrogenated and hydrogenated using PyMOL Version 2.0, and the compound ligands were converted to PDBQT format using the 3D SDF files, and finally, the PDB files of receptors and ligands were converted to PDBQT format using MGLTOOLS version 1.5.7. Subsequently, proteins were used as receptors and small molecules as ligands, and the active pockets with the highest drugScore scores were selected as the active sites for molecular docking based on the (https://proteins.plus/) prediction results, and the binding capacity was predicted using AutoDock Vina software.

### Cell culture and grouping

Human GC cell lines, including AGS and HGC27 purchased from Pricella Biotchnology Co., Ltd (Wuhan, China), were used in this study. F12K medium, supplemented with fetal bovine serum (FBS, 10%) and 1% streptomycin and penicillin (Gibco, MA, USA), was utilized for the culture of AGS cells. HGC27 cells were cultured in minimum essential medium (MEM), which was supplemented with 1% streptomycin and penicillin, as well as 10% FBS. All the cells were incubated in a humidified incubator 5% CO_2_ at 37 °C.

**Fig. 1:**
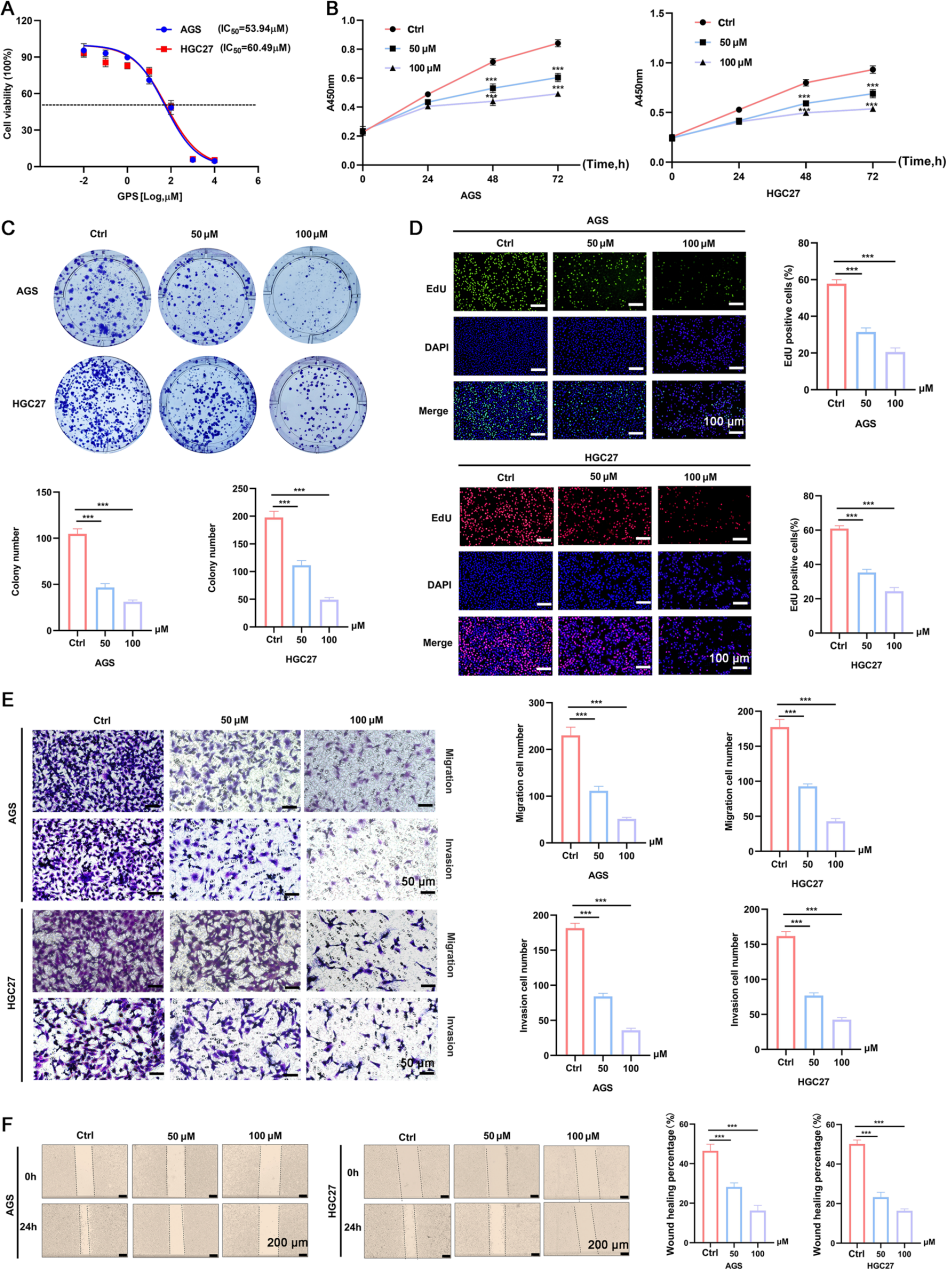
GPS inhibited the cellular proliferation, migration and invasion of AGS and HGC27 cells in a dose-dependent manner. **A** Selection of IC_50_ of GPS in two cell lines. **B** CCK-8 assay, **C** Colony formation assay, and **D** EdU staining kit were performed to measure proliferation ability of GC cells. **E** Transwell assay and **F** Wound healing assay were performed to measure migration and invasion ability of GC cells. (***P < 0.001)

For the grouping, both AGS and HGC27 cells were divided into three groups, including control group, and GPS treatment groups, subjected to GPS with a concentration of 50 µM and 100 µM (Selleckchem, Houston, TX, USA), respectively. The selection of GPS concentrations utilized in this study was decided based on the IC_50_ test (Fig. 1A).

### Cellular viability assay

AGS or HGC27 cells (1 × 10^4^) were seeded into 96-well plates. Cells were given GPS treatment with a cellular density of reaching 60% or more at 0, 24 h, 48 h and 72 h, respectively. Then, CCK-8 commercial kit was utilized for the evaluation of cellular viability, according to the previous description [14]. After incubating for 2 h, the cellular viability was measured with a microplate reader at an absorbance wavelength of 450 nm.

### Colony formation assay

Cells (1 × 10^3^) were cultured for 12 days in a 6 cm petri dish. Then, the cells were washed with PBS, followed by fixation with precooled methanol. The colony formation was counted after staining with crystal violet for 20 min.

### EdU staining

Commercial EdU staining kit purchased from Beyotime (Shanghai, China) was utilized for the evaluation of cellular proliferation, according to the manufacturer’s instructions. Cells (1 × 10^4^) in each group were incubated with EdU buffer (10 μM) at 37 ℃ for 2 h. Then, the supernatant was discarded and washed with PBS twice, followed by fixing with paraformaldehyde for 20 min. Afterwards, the mixture was incubated with freshly prepared cocktail buffer. For the calculation of proportion of EdU-positive cells, the number of fluorescent stained cells was divided by the number of cells stained with DAPI.

### Transwell assay

Transwell assay was conducted to evaluate the migration and invasion of AGS and HGC27 cells. For the migration assay, cells (4 × 10^4^) suspended in serum-free medium (100 μL) were added to the upper chamber. The lower chamber was added with medium (750 μL) containing 10% FBS, to induce the migration of cells. The invaded cells were washed with PBS thrice, followed by fixation for 30 min using 4% paraformaldehyde and staining with 1% crystal violet. For the invasion assay, the matrigel was added to the upper chamber. The other procedures were similar to those of the migration assay. The images were observed under a magnification of 100 × .

### Wound healing assay

Cells (6 × 10^5^) were cultured on 6-well plates. Then, we scratched the plate bottom from top to bottom with a pipettor. The free cells were washed away with PBS buffer. The migration distance was observed at 0 and 24 h, respectively. The area for the wound healing was determined with IMAGE J software.

### Flow cytometry

The apoptosis in each cell line was determined with the commercial Annexin V: FITC Apoptosis Detection Kit I (BD Biosciences, USA). Cells (5 × 10^5^) were seeded in 6-well plates, and were resuspended in 1 × binding buffer. Afterwards, the mixture was stained with Annexin V-Alexa Fluor647 (50 µg/mL) and propidium iodide (10 µg/mL) for 30 min. Finally, the cellular apoptosis was determined using flow cytometry.

### Transmission electron microscope (TEM)

Cells (4 × 10^5^) were seeded on 6-well plates, and were treated with GPS until a confluence of 60%. The samples were fixed, dehydrated and permeated successively. Resin blocks were cut into ultrathin sections (80 nm). The sections were stained with uranium acetate, followed by observing under a JEM-1200EX II electron microscope (Tokyo, Japan).

### Determination of mitochondrial membrane potential (MMP)

MMP was determined using the commercial JC-1 kit (MK3204, MaokangBio, Shanghai, China), according to the manufacturer’s instructions. In brief, the cells in each group were washed using PBS twice, and mixed with the JC-1 buffer at 37 ℃ for 30 min. Finally, the mixture was washed using 1 × JC-1 staining buffer and observed under a fluorescent microscope.

### Western blot

Total protein from AGS and HGC27 cells was extracted using commercial kit, and the protein concentration was determined with BCA method. The samples were subjected to SDS–PAGE, and were transferred to the PDVF membrane that were placed in 5% BSA solution (ThermoFisher Scientific, MA, USA). The primary antibodies were incubated at four degrees overnight. The primary antibodies utilized in the experiments included anti-BAX (Proteintech, 50599-2-Ig, 1:1000), anti-β-action (Proteintech, 66009-1-Ig, 1:5000), anti-Bcl-2 (Proteintech, 68103-1-Ig, 1:1000), anti-PARP (Cell Signaling Technology, #9532, 1:1000), anti-Cleaved PARP (Cell Signaling Technology, #5625, 1:1000), anti-Caspase-3 (Cell Signaling Technology, #14220, 1:1000), anti-Cleaved Caspase-3 (Cell Signaling Technology, #9664, 1:1000), anti-PI3K (Affinity, AF6241, 1:1000), anti-p-PI3K (Affinity, AF3242, 1:1000), anti-AKT (Cell Signaling Technology, #4691, 1:1000), anti-p-AKT (Cell Signaling Technology, #4060, 1:1000), anti-EGFR (Proteintech, 66455-1-Ig, 1:1000), Anti-Cyt C (Proteintech, 10993-1-AP, 1:1000). The secondary antibodies were added and incubated at room temperature for 1 h. Finally, the bands were visualized using the IMAGE J software, with β-action serving as the internal standard.

### Animal, modeling process and grouping

BALB/c nude mice (male, 6 weeks) were purchased from Vital River (Beijing, China) were subjected to induction of xenograft tumor model. Briefly, HGC27 cells (5 × 10^6^) were given via subcutaneous injection to the position near the right groin. Animals with tumor mass on day 7 were randomly divided into control group (n = 8) and GPS group (n = 8), treating with PBS and 40 mg/kg GPS via intraperitoneal injection, respectively. The PBS or GPS was given once per 3 days. The volume of the mass and body weight of mice were measured every 3 days. On day 28, the animals were euthanized and the tumor tissues and organs were collected for the following analysis. The study protocols were approved by the Ethical Committee of the First Affiliated Hospital of Shandong First Medical University (Approval No.: 2021-S1039).

### Induction of pulmonary metastasis model

To investigate the potential effects of GPS on the metastasis of GC, we first established the pulmonary metastasis model by tail vein injection of HGC27 cells (3 × 10^6^) with expression of luciferase. Subsequently, the mice were given intraperitoneal injection of GPS (40 mg/kg) or PBS on day 2, the PBS or GPS was given once per 3 days (5 mice in each treatment). On day 28, d-fluorescein (100 mg/kg) was given via intraperitoneal injection after anesthesia, followed by observing the tumor size after in vivo imaging. Finally, the animals were sacrificed to obtain the pulmonary tissues.

### IHC and HE staining

Tumor tissues were fixed using paraformaldehyde and were embedded in paraffin. The sections were incubated at 70 ℃ and dewaxed in xylene, followed by rehydration in ethanol. Upon heating in a high pressure for 10 min, the sections were treated with sodium citrate buffer (10 mmol/L, pH = 6.0). To inhibit the activity of endogenous peroxidase, the mixture was exposed in 3% hydrogen peroxide for 30 min at 37 ℃. Then, the mixture was blocked in goat serum for 30 min, and incubated with the primary antibodies including overnight including anti-EGFR (Proteintech, 66455-1-Ig, 1:300), anti-PI3K (Affinity, AF6241, 1:100), anti-p-PI3K (Affinity, AF3242, 1:100), anti-AKT (Cell Signaling Technology, #4691, 1:200), anti-p-AKT (Cell Signaling Technology, #4060, 1:100), anti-Ki67 (Proteintech, 27309-1-AP, 1:1000). Then, the HRP-conjugated secondary antibodies were given at 37 °C for 30 min, together with observation under a microscope. For the HE staining, sections (5 μm) were dewaxed and dehydrated, followed by incubating with hematoxylin at room temperature for 5 min. The sections were incubated with hydrochloric acid alcohol (1%) for 30 s, and were dipped in eosin for 2 min. After dehydration, the sections were sealed with neutral resin and observed under a microscope according to the previous description [15].

### Statistical analysis

All the tests including the cellular experiments and animal studies were performed at least in triplicate. The data were presented as mean ± standard deviation. Data analysis was performed using SPSS23.0 software. The figures were drawn using the Prism GraphPad software (version 8.0). A P value of less than 0.05 was considered as statistical difference.

## Results

### GPS inhibited the proliferation, invasion and migration of GC cell lines

GPS could significantly inhibit the cellular proliferation compared with that of control group (Fig. 1B). In addition, the colony formation in the GPS group showed significant decrease compared with that of the control group (Fig. 1C), showing a dose-dependent manner. EdU staining indicated that the proportion of EdU positive cells in the GPS groups showed significant decrease compared with that of control group (Fig. 1D). Similarly, the transwell assay and wound healing assay confirmed the inhibitory effects of GPS on the migration and invasion of AGS and HGC27 cells (Fig. 1E, F). Taken together, these data suggested that GPS inhibited GC cells proliferation, invasion and metastasis in a concentration-dependent manner.

### GPS induced GC cells apoptosis

**Fig. 2:**
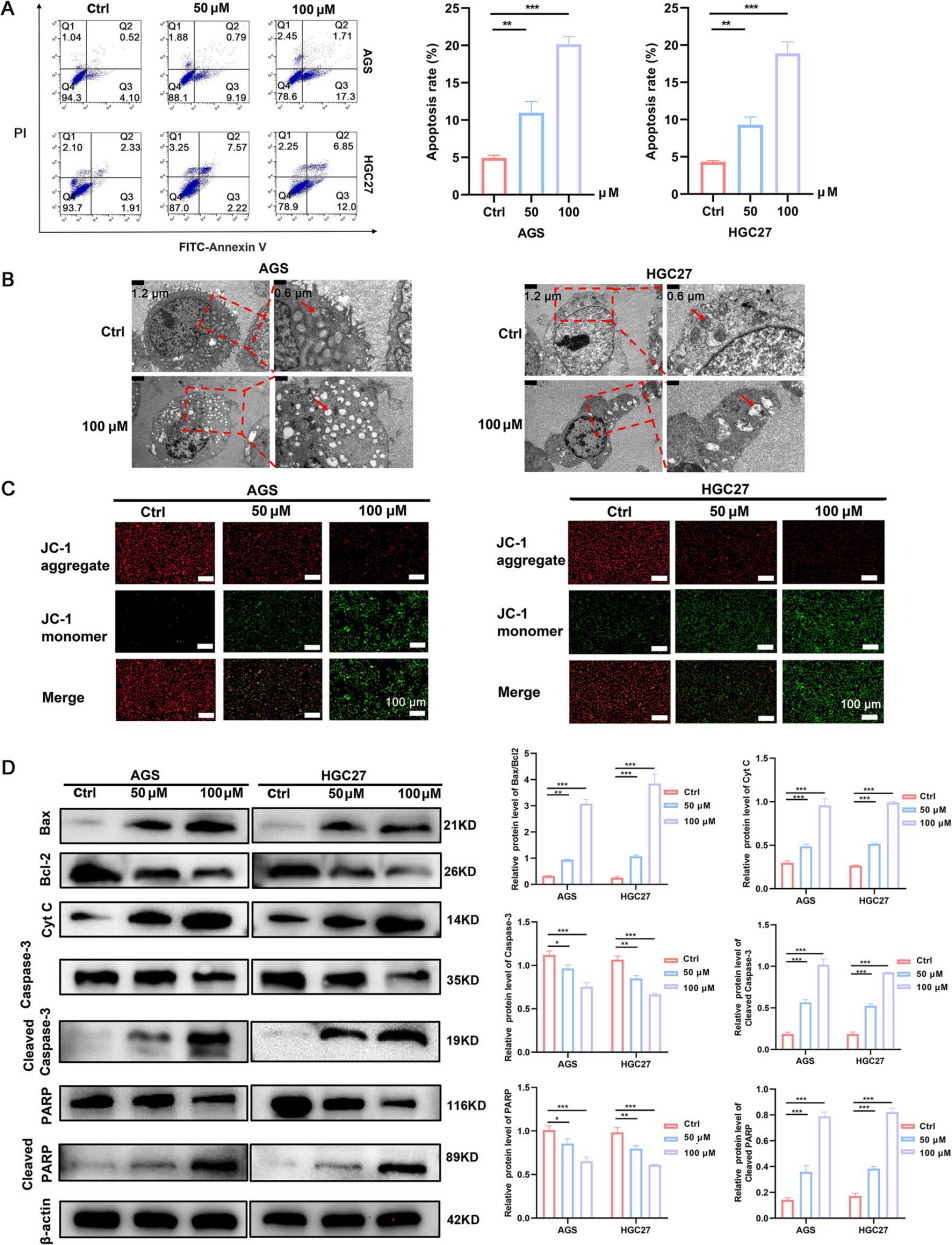
GPS promoted the cellular apoptosis, which was related to the rupture of mitochondrial integrity. **A** GPS induced cellular apoptosis in AGS and HGC27 cells. **B** TEM showed that GPS induced rupture of mitochondrial structure. **C** GPS induced decrease of JC-1 aggregate and increase of JC-1 monomer in cells. **D** GPS regulated the expression of apoptosis-related proteins (*P < 0.05, **P < 0.01, ***P < 0.001)

Flow cytometry indicated that GPS induced apoptosis in a dose-dependent manner (Fig. 2A). Mitochondrial dysfunction can trigger intracellular signaling cascades that initiate programmed cell death, leading to apoptosis in a variety of tumour cells [16]. To further validate whether GPS could induce mitochondrial apoptosis, TEM was conducted to measure the mitochondrial injury after GPS treatment. As shown in Fig. 2B, GPS triggered mitochondrial injuries featured by excessive mitochondrial swelling and rupture of mitochondrial cristae. Besides, GPS triggered the decrease of MMP in AGS and HGC27 cells, featured by significant decrease of JC-1 aggregates and significant increase of JC-1 monomers (Fig. 2C). In addition, Western blot analysis was performed to determine the expression of apoptosis-related proteins. As shown in Fig. 2D, GPS could induce significant downregulation of the anti-apoptotic Bcl-2. In addition, it significantly induced the up-regulation of the pro-apoptotic proteins Cleaved Caspase-3, Cleaved PARP, Bax and Cyt C. Taken together, these results suggest that GPS-induced apoptosis is mediated by mitochondrial dysfunction, thereby activating the mitochondrial apoptotic pathway in GC cells.

### Network pharmacological analysis predicts GPS targets and pathways affecting GC

**Fig. 3:**
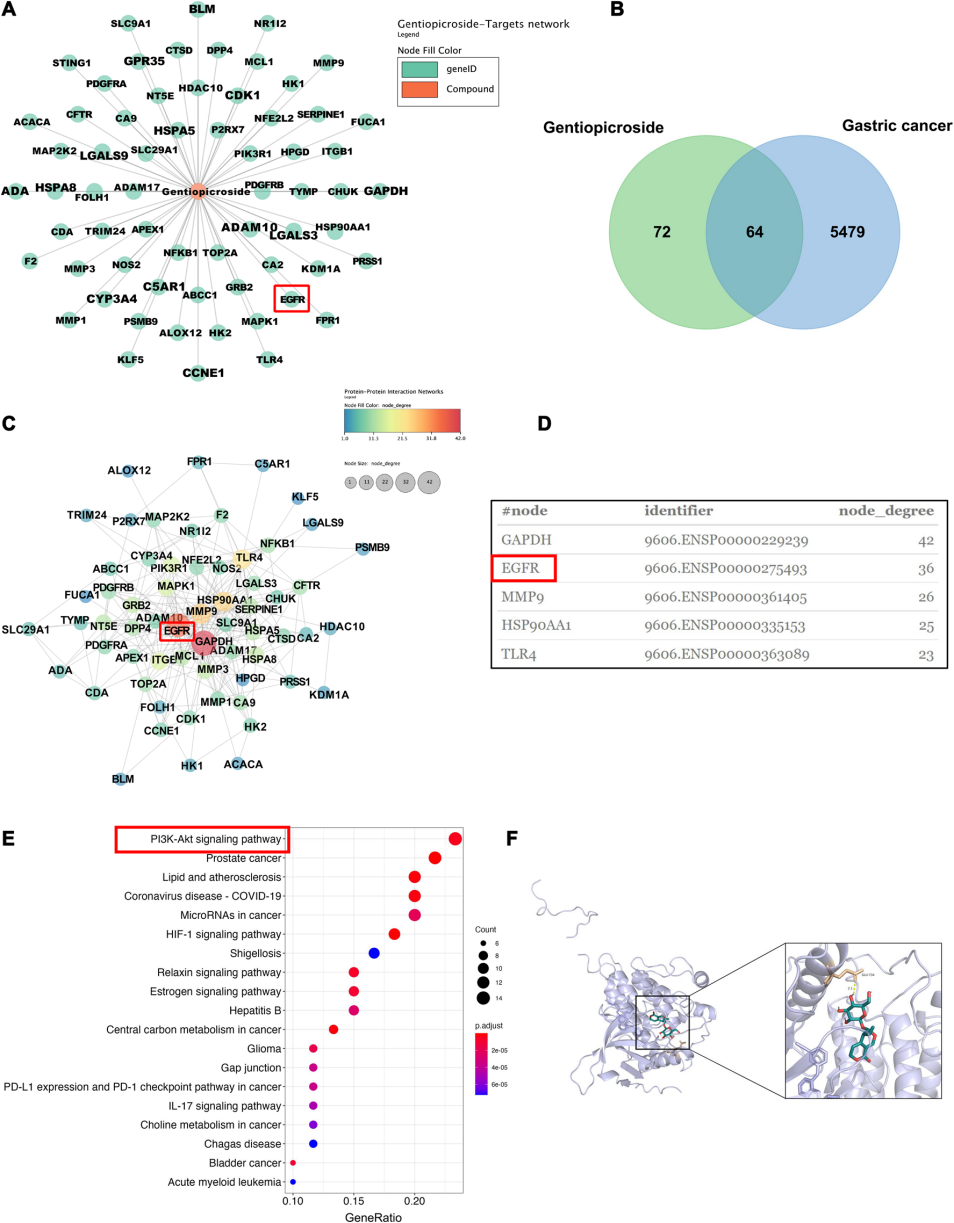
Network pharmacological analysis predicted GPS targets and pathways affecting GC. **A** Predicting the intersection of targets of action of GPS. **B** Venn diagram of the cross-targets associated with the target of GC and the target of GPS. **C** PPI network of Gentianoside on GC targets. **D** List of hub genes for GPS on GC by degree method. **E** Analysis of KEGG enrichment of GC target genes by GPS. **F** Molecular docking analyses of the three-dimensional structure of GPS and EGFR

Through taking the intersection of the action targets obtained from the prediction of GPS by SwissTargetPrediction (Fig. 3A) and the action targets obtained from the retrieval of OMIM and Genecards database for GC, a total of 64 cross-targets potentially related to the treatment of GC by GPS were identified (Fig. 3B). In order to identify the core targets of GPS for GC treatment, we used STRING to construct the cross-targets of the PPI network and analysed the results using Cytoscape. The PPI network is shown in Fig. 3C. The top 5 interacting hub proteins were obtained based on the basis of the high and low *p* value (Fig. 3D). The related KEGG pathways were listed according to the ascending order of *p* value (Fig. 3E). The results showed that the PI3K-AKT signaling pathway had the highest significance and the highest number of enriched genes. Further analysis of the top five scoring hub proteins in the PPI network revealed that EGFR was the highest scoring protein excluding the internal reference protein GAPDH. Intriguingly, molecular docking analyses of the three-dimensional structure of GPS and EGFR showed that there is an interaction between GPS and EGFR (Fig. 3F). Based on a large number of previous studies suggesting that EGFR/PI3K/AKT are important signals that regulate cell proliferation and apoptosis [17], we deduced that the EGFR/PI3K/AKT axis may be the key to the function of GPS in GC.

### GPS inhibited the activation of EGFR/PI3K/AKT

**Fig. 4:**
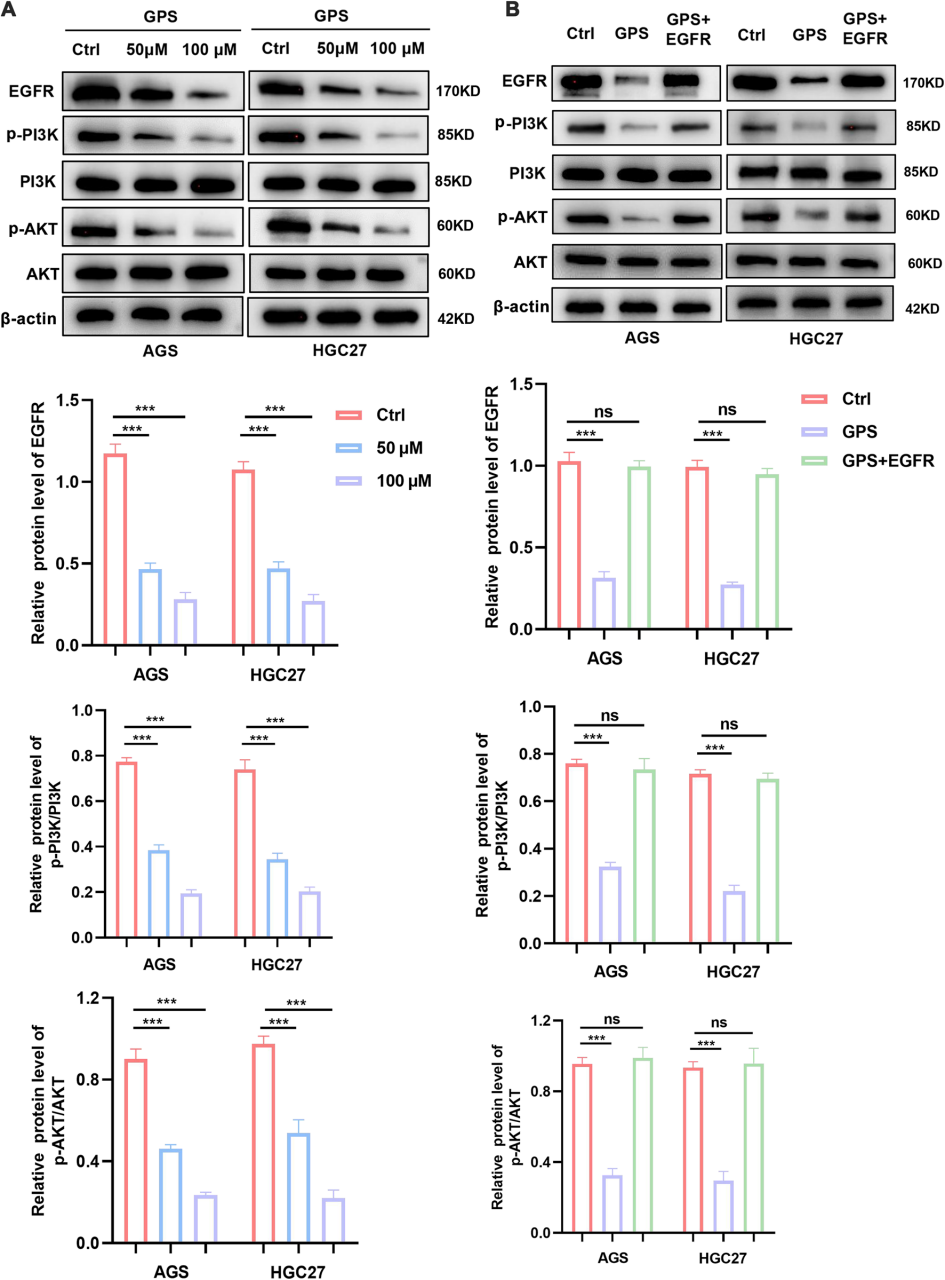
GPS inhibited the activation of EGFR/PI3K/AKT. **A** The protein expression levels of PI3K/AKT signaling in GPS-treated GC cells were determined by Western blot. **B** The protein expression levels of PI3K/AKT signaling in GPS-treated GC cells after addition of overexpressed EGFR plasmid were determined by Western blot. (***P < 0.001; ns, non-significant)

EGFR/PI3K/AKT signaling pathway plays an important role in cell proliferation, migration and apoptosis. EGFR stimulates tumour growth by modulating dimerisation of Ras proteins, which leads to a phosphorylation cascade that activates the PI3K/AKT signaling pathway [18]. Therefore, to confirm whether EGFR was regulated in GPS-treated GC cells, we found by Western blot assay that EGFR levels in GC cells decreased in a concentration-dependent manner after GPS treatment (Fig. 4A). At the same time, we also detected the expression levels of proteins related to PI3K/AKT signaling pathway, one of the downstream pathways of EGFR. As shown in the figure, GPS had a significant dose-dependent inhibitory effect on the phosphorylated expression of PI3K and AKT in GC cells, whereas the total PI3K and AKT protein levels remained essentially unchanged.

To further validate the relationship between GPS and EGFR, we added an overexpression plasmid of EGFR (Sequence: NM-005228) in GC cells treated with GPS, and then detected the protein level of this pathway (Fig. 4B). The analysis showed that the addition of overexpressed EGFR significantly attenuated the inhibitory effect of GPS on the phosphorylation of PI3K and AKT downstream of the EGFR. These results suggest that GPS inhibits the PI3K/AKT pathway by reducing EGFR expression.

### GPS suppressed malignant behaviour of GC cells by inhibiting the EGFR/PI3K/AKT axis

**Fig. 5:**
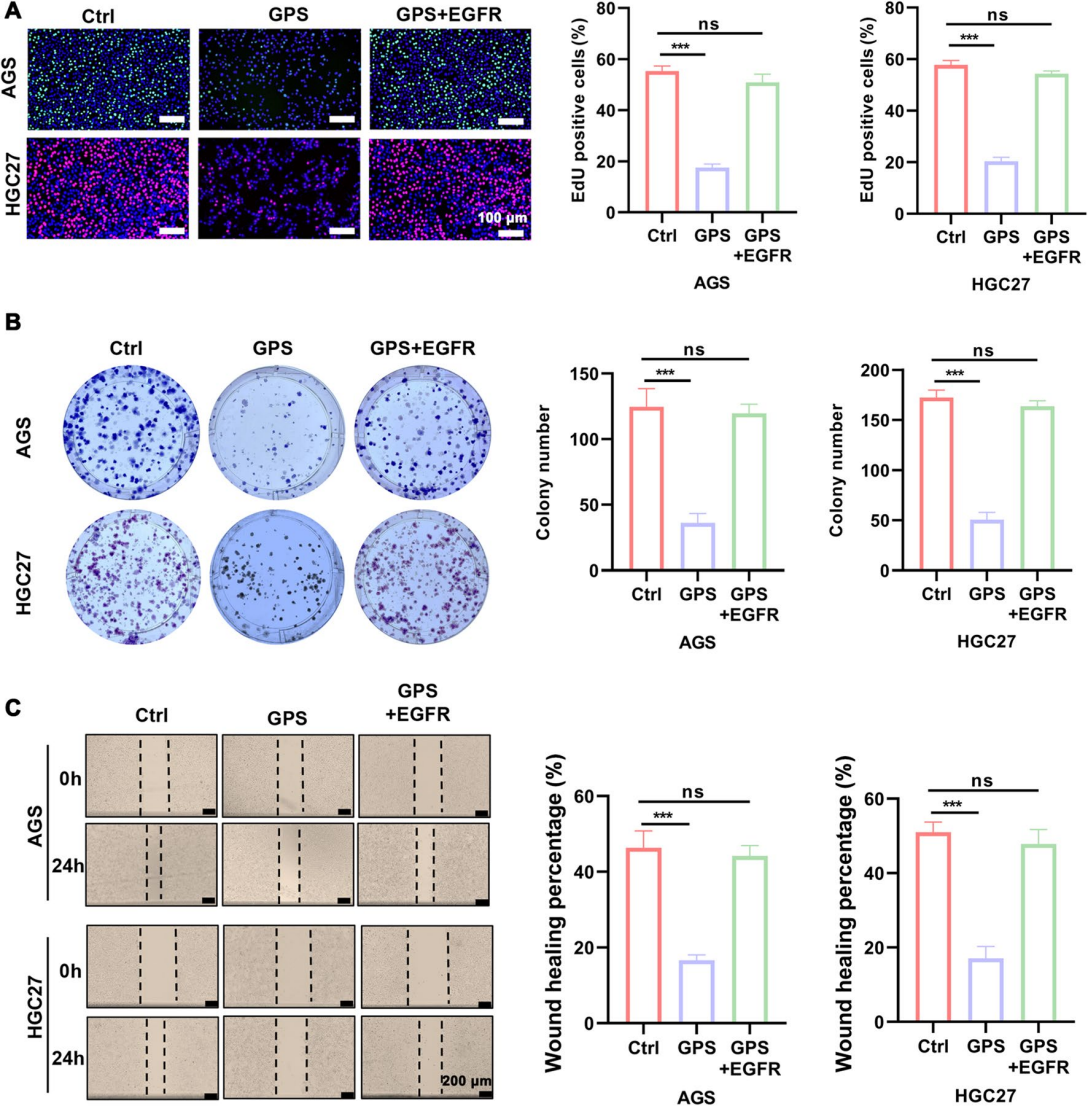
GPS inhibited GC cells proliferation and migration by inhibiting the EGFR/PI3K/AKT axis. **A** EdU staining kit, **B** Colony formation assay and **C** wound healing assay were performed to measure the proliferation and migration ability of GC cells treated with GPS (100 μM) and GC cells treated with GPS (100 μM) co-overexpressing EGFR plasmid. (***P < 0.001; ns, non-significant)

**Fig. 6:**
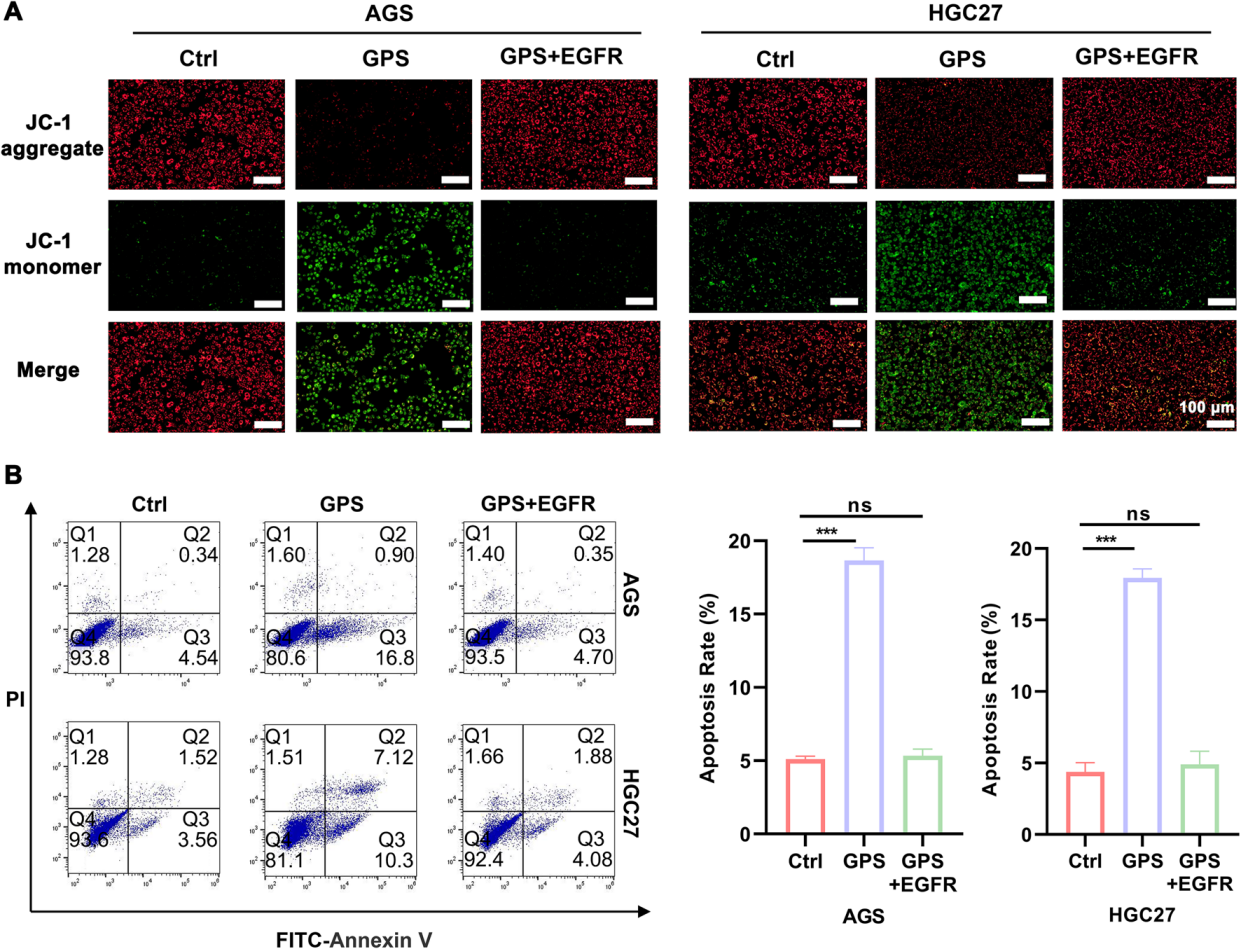
GPS promoted GC cells apoptosis by inhibiting the EGFR/PI3K/AKT axis. **A** The tendency of GPS (100 μM)-induced decrease in JC-1 aggregates and increase in JC-1 monomers in GC cells could be reversed after co-application of overexpressing EGFR plasmid. **B** The tendency of GPS (100 μM) to promote apoptosis in GC cells could be reversed after co-application of overexpressing EGFR plasmids. (***P < 0.001; ns, non-significant)

To verify whether GPS affects biological behaviours such as proliferation and apoptosis of GC cells by inhibiting EGFR/PI3K/AKT, we performed rescue experiments by adding overexpression plasmid of EGFR to GPS (100 μM)-treated cells. It was found by EdU as well as colony formation assay that overexpression plasmid of EGFR-treated GC cells partially reversed the proliferation inhibition induced by GPS (Fig. 5A, B). It was also seen by wound healing assay that the GPS-induced diminished migration ability of GC cells was also partially restored after the application of overexpression plasmid of EGFR (Fig. 5C). In the JC-1 mitochondrial membrane potential assay, the GPS-induced decrease in MMP in overexpression plasmid of EGFR-treated GC cells was also suppressed (Fig. 6A). The results of apoptosis detection by flow cytometry also showed that overexpression plasmid of EGFR treatment partially reversed the apoptosis induced by GPS in GC cells (Fig. 6B). From the above results, we conclude that GPS inhibits the malignant behaviour of GC cells by inhibiting EGFR/PI3K/AKT.

### GPS inhibited the tumor growth and metastasis in vivo

**Fig. 7:**
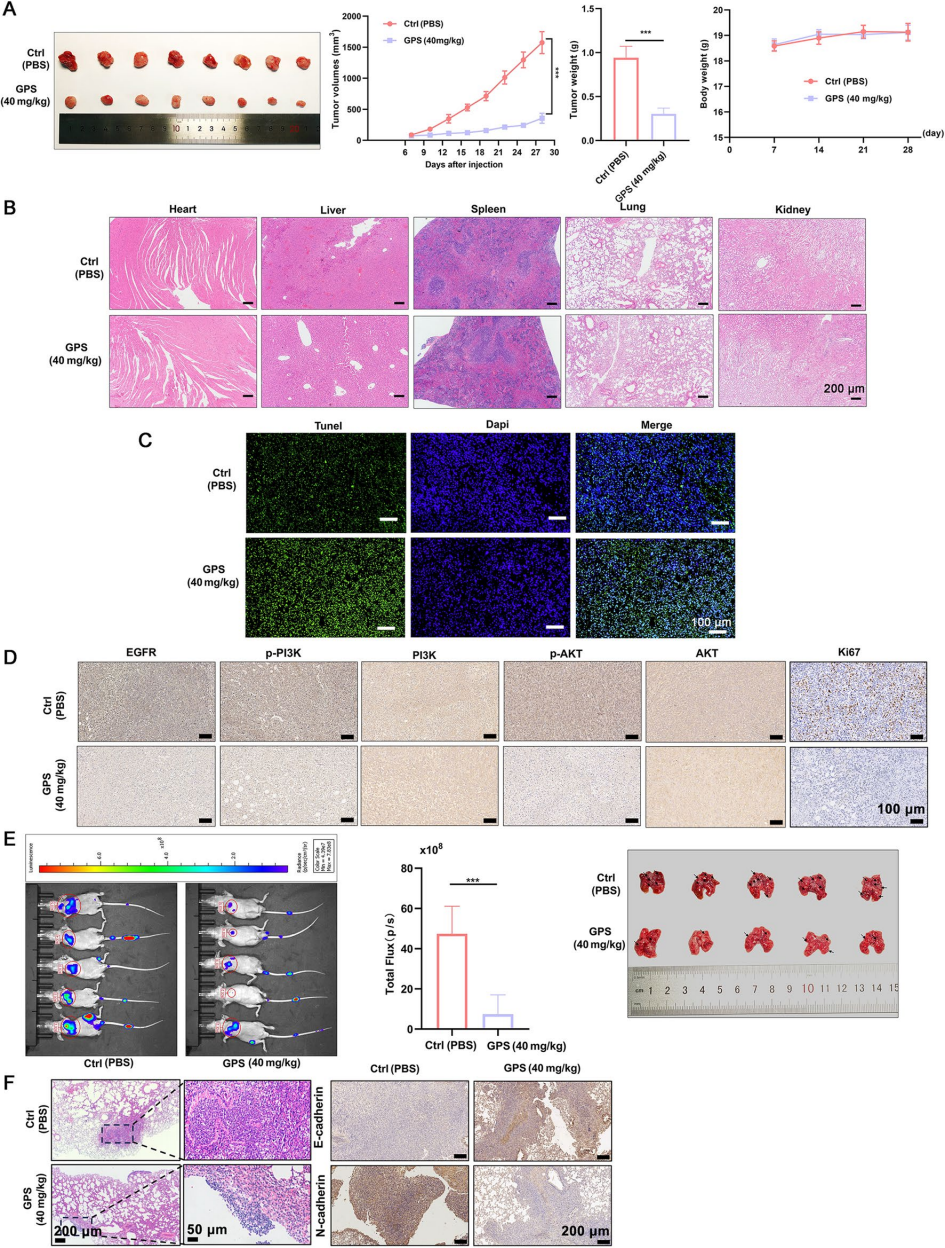
GPS inhibited the tumor growth and metastasis in vivo. **A** Tumor volume, tumor weight (day 28) showed significant decrease in the GPS group compared with control group, without changing the body weight. **B** GPS showed no cytotoxicites in heart, liver, spleen, lung and kidney tissues. **C** GPS promoted the apoptosis of cancer cells in vivo. **D** IHC showed that GPS could down-regulate the expression of EGFR, p-PI3K and p-AKT proteins and inhibited the proliferation of cancer cells in vivo. **E, F** GPS induced pulmonary metastasis, and significant up-regulation of E-cadherin and down-regulation of N-cadherin (***P < 0.001)

The tumor weight and tumor volume in the GPS group showed significant decrease compared with those of control group (Fig. 7A). In contrast, the body weight of the mice showed no differences between the GPS and control groups. For the safety concerns, GPS caused no obvious toxicities to the heart, liver, spleen, lung and kidney tissues compared with control group (Fig. 7B). GPS treatment, under in vivo conditions, enhanced the apoptosis of tumor cells as revealed by TUNEL assay (Fig. 7C). Consistently, the Ki67 positive cells presenting the cancer cell proliferation in vivo was significantly decreased in number after GPS treatment. IHC indicated GPS down-regulated the expression of EGFR, p-PI3K and p-AKT (Fig. 7D). All these indicated that GPS inhibited the proliferation of tumor cells without inducing obvious toxicities, which may validate the results of in vitro data that GPS could modulate the EGFR/PI3K/AKT signaling pathway.

After intraperitoneal injection of D-fluorescein, in vivo imaging was performed to monitor the pulmonary metastasis of GC cells (Fig. 7E). GPS treatment could significantly attenuate the pulmonary metastasis featuring by decreased number and size of lung nodules compared with control group, together with significant up-regulation of E-cadherin and down-regulation of N-cadherin (Fig. 7F).

## Discussion

In recent years, research on natural products for the treatment of cancer has received increasing attention [19]. GPS, isolated from Gentianella acuta, has been shown to exert pharmacological activities including ameliorating diabetic nephropathy, reducing pain, pruritusp, as well as antidepressant-like effects [20]. Recently, a few studies have paid close attention to its potential roles in the treatment of cancer, such as liver cancer and cervical cancer [9]. Very rare studies have focused on the molecular mechanisms which GPS induces and the resulting changes in cellular phenotypes. Among these studies, GPS has been reported to show tumor-suppressing effects [11], but the exact mechanisms of how GPS inhibited cancer growth are still not well defined. Our data reported for the first time that GPS inhibits GC malignant progression by targeting the EGFR/PI3K/AKT signaling pathway.

EGFR, a member of the EGFR/ErbB subfamily of receptor tyrosine kinases (RTKs) , plays a crucial role in the development of various cancers [21], and it promotes other cellular processes, such as cell proliferation, survival, migration/inhibition of apoptosis [22, 23]. The PI3K/AKT/mTOR signaling pathway is one of the downstream signaling pathways of EGFR, which is closely related to the regulation of proliferation and apoptosis [24]. Reduced expression of EGFR inhibits the activity of the PI3K/AKT signaling pathway, which in turn reduces the proliferation and metastatic capacity of the cells [25], thereby inducing a series of downstream signaling events [26]. The PI3K-AKT pathway is a negative regulator of apoptosis. Once PI3K-AKT signaling is over-activated, cell proliferation and migration are promoted and lead to inhibition of apoptosis [27]. In this study, we first treated GC cell lines with GPS and showed that GPS significantly inhibited the proliferation, migration and invasion of GC cell lines, while significantly inducing apoptosis in these cells. Apoptosis is an active and programmable process of cell death [28]. It can be divided into endogenous apoptosis and exogenous apoptosis [29]. In addition, the mitochondrial apoptotic pathway, as a major regulator of endogenous apoptosis, is involved in the increased outer membrane permeability, changes in MMP and Cyt C release [30]. This study shows that GPS induces a decrease in MMP and the release of Cyt C. Dysfunction of mitochondria may be the cause of apoptosis in GC cells due to GPS. GPS leads to the release of Cyt C from the inner mitochondrial membrane into the cytoplasm and interacts with apoptotic proteins, which further initiates a cascade of reactions to activate downstream apoptotic execution proteins [31]. In this study, the detection of apoptosis-related proteins in GPS-treated GC cells revealed that the expression level of Bcl-2 was significantly reduced, while the expression levels of Bax, Cleaved Caspase-3, and Cleaved PARP were significantly increased. All these results indicated that GPS induced apoptosis in GC cells through the mitochondria-associated apoptosis pathway. To explore the underlying mechanisms, we found that GPS was highly correlated with EGFR through network pharmacology enrichment analysis and molecular docking analysis. Subsequently, we synthesized plasmids for EGFR overexpression. The data showed that in the presence of EGFR overexpression, the anticancer effect of GPS was reversed, which was mainly manifested by the up-regulation of proliferation-associated proteins and the down-regulation of apoptosis-associated proteins. This implies that GPS exerts its anticancer effects by inhibiting EGFR, thereby enhancing apoptosis and inhibiting proliferation in GC cell lines.

In the present study, we hypothesized that GPS may inhibit the PI3K/AKT signaling pathway by suppressing the expression of EGFR. Western blot analysis showed that GPS downregulated the expression of EGFR, p-PI3K, and p-AKT in GC cells in a dose-dependent manner. This implies that GPS can significantly inhibit the PI3K/AKT signaling pathway in vitro. When overexpressing EGFR plasmid was used, the inhibitory effect of GPS on the PI3K/AKT signaling pathway was reversed. This verified our hypothesis that GPS inhibited the PI3K/AKT signaling pathway by suppressing EGFR expression. In vivo experiments showed that GPS inhibited tumor proliferation and lung metastasis and promoted apoptosis. Meanwhile, IHC showed that EGFR, p-PI3K and p-AKT proteins were significantly down-regulated compared with the control group. Regarding safety, our data show that GPS did not produce significant cytotoxicity in animals. All this confirms that GPS is promising in killing cancer cells in vivo and does not cause severe cytotoxicity to normal cells. In summary, we concluded that GPS inhibited the EGFR/PI3K/AKT signaling pathway to suppress GC progression.

This study does have some limitations. This is a preliminary study on the role of GPS in GC. So far, we have only studied how GPS regulates the EGFR/PI3K/AKT signaling pathway under in vitro and in vivo conditions. More signaling pathways, such as the MAPK and P53 pathways, and how GPS inhibits EGFR expression have not been studied by us, but this does not hamper the value of our research. In the near future, we will focus on the above issues.

## Conclusions

GPS could inhibit GC malignant progression by targeting the EGFR/PI3K/AKT signaling pathway. GPS could inhibit the growth of tumor and pulmonary metastasis in vivo, without causing obvious cytotoxicity.

## Data Availability

The data sets generated during and/or analysed during the current study are available from the corresponding author on reasonable request.

## Abbreviations

EGFR: Epidermal growth factor receptor
FBS: Fetal bovine serum
GC: Gastric cancer
GPS: Gentiopicroside
IHC: Immunohistochemistry
MEM: Minimum essential medium
MMP: Mitochondrial membrane potential
PPI: Protein interaction
TCM: Traditional Chinese medicine
TEM: Transmission electron microscope
